# Artificial neural network - an effective tool for predicting the lupus nephritis outcome

**DOI:** 10.1186/s12882-022-02978-2

**Published:** 2022-11-28

**Authors:** Jakub Stojanowski, Andrzej Konieczny, Klaudia Rydzyńska, Izabela Kasenberg, Aleksandra Mikołajczak, Tomasz Gołębiowski, Magdalena Krajewska, Mariusz Kusztal

**Affiliations:** Department of Nephrology and Transplantation Medicine, Wroclaw Medical University, Borowska 213, 50-556 Wroclaw, Poland

**Keywords:** Artificial intelligence, Machine learning, Proteinuria, Systemic lupus erythematosus, Lupus nephritis, End-stage renal disease

## Abstract

**Background:**

Lupus nephropathy (LN) occurs in approximately 50% of patients with systemic lupus erythematosus (SLE), and 20% of them will eventually progress into end-stage renal disease (ESRD). A clinical tool predicting remission of proteinuria might be of utmost importance. In our work, we focused on predicting the chance of complete remission achievement in LN patients, using artificial intelligence models, especially an artificial neural network, called the multi-layer perceptron.

**Methods:**

It was a single centre retrospective study, including 58 individuals, with diagnosed systemic lupus erythematous and biopsy proven lupus nephritis. Patients were assigned into the study cohort, between 1st January 2010 and 31st December 2020, and eventually randomly allocated either to the training set (N = 46) or testing set (N = 12). The end point was remission achievement. We have selected an array of variables, subsequently reduced to the optimal minimum set, providing the best performance.

**Results:**

We have obtained satisfactory results creating predictive models allowing to assess, with accuracy of 91.67%, a chance of achieving a complete remission, with a high discriminant ability (AUROC 0.9375).

**Conclusion:**

Our solution allows an accurate assessment of complete remission achievement and monitoring of patients from the group with a lower probability of complete remission. The obtained models are scalable and can be improved by introducing new patient records.

## Background

Among patients suffering from systemic lupus erythematous (SLE), almost all of them have, to some extent, a renal affection during the disease course, and between 40% and 70% will develop clinically diagnosed renal involvement named lupus nephritis (LN) [1]. It is a major risk factor of morbidity and mortality in SLE, and 10% of patients with LN will eventually develop end-stage renal disease (ESRD), within 5 years of disease onset [2].

Renal biopsy is the gold standard for LN diagnosis. Based on kidney biopsy assessment, a patient can be classified into any of six histological categories, according to the International Society of Nephrology/Renal Pathology Society classification, of which classes III–VI are associated with the highest risk of long-term damage[3]. Class VI reflects the most advanced stage, where patients require any type of renal replacement therapy, including kidney dialysis or transplantation [3]. Subsequential treatment decisions are based on glomerular involvement. Unfortunately, current standards for diagnosis and treatment of LN are unsatisfactory and it is neither possible to accurately predict a response to therapy nor the long-term outcome for individual patients [4]. Therefore, there is a need for establishing of predictive models allowing estimation of long-term results. Currently available studies provide several both clinical and histopathological factors, related to unsatisfactory results. Among them, the most crucial predictors of poor outcome are male gender, younger age, hypertension, increased serum creatinine, African American race, proliferative disease, high activity and chronicity index, glomerulosclerosis and crescents, interstitial inflammation, tubular injury, and an extent of interstitial fibrosis [5]. Achievement of a proteinuria < 0.7 g/day at month 12, best predicts good outcome at 7 years and inclusion of haematuria at month 12 undermines the sensitivity of early proteinuria decrease for the prediction of good outcome [6].

Based on the clinical data derived from patients with diagnosed LN and using artificial intelligence techniques, and artificial neural networks, we have built a machine learning model allowing prediction of complete remission in a patient with LN.

## Methods

### Data collection

It was a single centre trial, including retrospective data of 58 patients with diagnosed systemic lupus erythematosus and biopsy-proven LN. The SLE diagnosis was based on EULAR/ACR classification criteria [7]. The following clinical parameters were included: age, gender, serum creatinine concentration, estimated glomerular filtration rate (eGFR) calculated by MDRD equation, C3 and C4 concentrations, serum albumin, extent of proteinuria measured as urine protein to creatinine ratio (UPCR), erythrocytes sedimentation rate (ERS), C-reactive protein (CRP) concentration, erythrocyturia assessed as number of red blood cells (RBC) on high-power field (HPF),

All parameters were collected at the time of kidney biopsy. Only patients with significant proteinuria (assessed as UPCR > 1.0 mg/mg) were included into study group. After 6 months of follow-up, a complete remission (CR) of LN was defined as UPCR < 0.5 and stable renal function, according KDIGO guidelines [8]. All patients were treated according to EURO-LUPUS regimen, using 6 intravenous pulses cyclophosphamide (500 mg each), followed by oral mycophenolate mofetil, unless contraindicated [9].

### Statistical scoring

The performance of the artificial neural network models was assessed with the following statistical indicators: area under the receiver-operator curve (AUROC), Accuracy, Precision, Recall and F1-Score. AUROC was used to assess the discriminant power of the artificial neural network.

### Artificial neural network

The entire project was created and run in the python 3.6.8 environment. Incomplete rows, containing blank cells, were removed from the original database, allowing reduction of the amount of available data, but got 100% complete dataset. In our previous work we analysed mostly random forest classifiers, due to their better performance against neural networks [10]. An artificial neural network is a complex structure consisting of several basic units, called artificial neurons. In its simplest form, there are perceptrons containing several inputs, with assigned weights and one output. Functions responsible for building a multi-layer perceptron came from the scikit-learn library. It is, to some extent, analogous to a biological neuron with many dendrites but only one axon. The interior of the perceptron is an activating function, superimposed on the sum of the products of the neuron’s inputs and the corresponding weights. The bias vector affects performance and results in better fitting to the data. Neurons are arranged in layers that are interconnected. In a multi-layer perceptron, these layers are organized in the input layer, hidden layers, and output neurons. Depending on the number of neurons and layers, different complexity may be obtained. Naturally, the greater the complication, there more of the possibilities of such network, but at the same time, the more time cost needed to train it.

$${\text{output}=\text{f}}_{\text{activation}}\left(\text{b}\text{i}\text{a}\text{s}+\sum _{\text{k}=1}^{\text{n}}{\text{i}\text{n}\text{p}\text{u}\text{t}}_{\text{k}}\bullet {\text{w}\text{e}\text{i}\text{g}\text{h}\text{t}}_{\text{k}}\right)$$

The activation function is analogous to the excitability threshold of a biological neuron. In MLP, this is a ReLu function that returns zero for all non-positive values and takes the input value for positive values.

$${\sigma }\left(\text{x}\right)=\text{max}\left(0,\text{x}\right)=\left\{\begin{array}{c}\text{x for positive values}\\ 0~\text{otherwise}\end{array}\right.$$

The activation function for the output in MLP is the logistic function, given by the following formula:

$$\sigma \left(x\right)=\frac{1}{1+{e}^{x}}$$

The complexity of the MLP neural network is related to the number of samples in the training set, the number of input features, predicted classes, and neurons in the respective layers. In mathematical notation it is written as O (n·m·o·h1·h2), where “n” is the number of samples in the training set, “m” is the number of input features, and “o” is the number of predicted classes. The sizes of the hidden layers are h1 and h2, respectively, and they denote the number of iterations leading to the best model.

The completed database has been recursively split into subsets per column. For example, the subsets contained data for all patients, but only for selected columns. The selection of input parameters was based on recursive searching of the subset space, individual evaluation of each statement, selection of hyperparameters and evaluation on the test set. Initially, we thought about applying heuristics to optimize models, but with a cut-off size of 1 to 45 neurons in the hidden layer, we did not experience an appreciable loss of resources, using brute force search. Naturally, we are aware that heuristics in model optimization are necessary in more advanced models and for larger input data. The search for optimization solutions for modelling in medicine can be an interesting subject of research and bring enormous progress in the field of personalized medicine. The main hyperparameters of the neural network are the number of neurons in the individual hidden layers. Due to the speed of calculations and their parallelism, we used a for-loop nested in the for-loop and limited the maximum number of neurons to 150 in a single layer. We are aware that the complexity was high, but in practice we were able to trace how the performance of the network changes depending on its structure, which, however, is not the subject of this work, but is discussed in another of our work [10]. The performance measured by AUROC, and Accuracy has been saved and finally the best configurations was chosen, allowing the most accurate prediction of total remission.

## Results

### Study population baseline characteristics

Retrospective data of 58 patients with biopsy proven LN, aged 18–72 years (36.05 ± 13.98), 48 women and 10 men, were included. All evaluated parameters and variables are presented in Table 1.

**Table 1:** Basic characteristics of the population

Parameter [N = 58]	Population (Mean ± Standard Derivation (SD) and range from minimal to maximal value) *Categorical (if applicable) ** Not included in program analysis
Gender (male/female)	10/48 (17.24%/82.76%)
Age at biopsy [years]	36.05 ± 13.98 (18 ÷ 72)
LN class II/III/IV/V/VI	1/12/25/12/8 (1.72%/20.69%/43.10%/20.69%/13.79%)
Number of glomeruli in specimen	20.26 ± 9.54 (6 ÷ 62)
Number of totally sclerotic glomeruli	1.88 ± 3.46 (0 ÷ 15)
Number of partially sclerotic glomeruli	3.81 ± 7.14 (0 ÷ 48)
Number of cellular/cellular-fibrotic crescents	1.23 ± 2.83 (0 ÷ 17)
Number of glomeruli with fibrillary necrosis	2.32 ± 4.42 (0 ÷ 25)
Number of glomeruli without changes	0.91 ± 2.28 (0 ÷ 12)
Activity Index	8.96 ± 4.51 (0 ÷ 22)
Chronicity Index	3.15 ± 2.13 (0 ÷ 8)
Interstitial fibrosis [%]	9.91 ± 7.16 (5 ÷ 40)
WBC [10^3/ul]	7.93 ± 3.02 (1.7 ÷ 15.57)
NEU [10^3/ul]	5.51 ± 2.49 (0.52 ÷ 12.84)
LYM [10^3/ul]	1.77 ± 0.93 (0.38 ÷ 4.95)
NLR	4.42 ± 4.36 (0.64 ÷ 24.05)
HGB [g/dl]	11.89 ± 1.73 (7.7 ÷ 17.1)
PLT [10^3/ul]	229.98 ± 78.65 (31 ÷ 475)
PLR	185.63 ± 144.51 (14.09 ÷ 831.58)
ERS [mm/1 h]	35.79 ± 23.44 (3 ÷ 128)
CRP [mg/l]	4.09 ± 7.86 (0.07 ÷ 43.69)
sCr [mg/dl]	1.28 ± 0.65 (0.6 ÷ 3.66)
eGFR [ml/min/1.73 m^2^]	65.72 ± 26.67 (13 ÷ 127)
TP [g/dl]	5.33 ± 0.93 (3.7 ÷ 7.5)
ALB [g/dl]	2.86 ± 0.56 (1.9 ÷ 3.9)
Total Cholesterol [mg/dl]	268.29 ± 82.39 (116 ÷ 578)
Triglycerides [mg/dl]	196.32 ± 106.88 (49 ÷ 541)
C3 [g/l]	0.84 ± 0.29 (0.32 ÷ 1.74)
C4 [g/l]	0.18 ± 0.10 (0.07 ÷ 0.42)
ANA (0/1/undefined)	3/48/7 (5.17%/82.76%/12.07%)
Erythrocyturia [RBC/HPF]	13/23/22 (22.41%/39.66%/37.93%)
UPCR [mg/mg]	3.29 ± 2.93 (0.5 ÷ 16.16)
Complete Remission [Yes/No]	18/40 (31.03%/68.97%)

Abbreviations. WBC – white blood cells; NEU - neutrophils, LYM – lymphocytes, NLR – neutrophil-to-lymphocyte ratio, HGB – haemoglobin, PLT - platelets, PLR – platelets-lymphocyte ratio, ERS – erythrocytes sedimentation rate, CRP – C-reactive protein, sCr – serum creatinine, eGFR – estimated glomerular filtration rate, ALB – serum albumin; TP – total protein, C3 – complement component 3, C4 – complement component 4, ANA – antinuclear antibodies, UPCR – urine protein-creatinine ratio,

The input database was randomly divided into training and testing cohorts. The characteristics of the divided groups are described in Table 2.

**Table 2:** Baseline characteristics of the patients enrolled in the cohorts. Training and testing groups are characterized by mean ± standard derivation (SD) and range from minimal to maximal value or as categorical, if applicable

Patients’ parameters [N]*	Study Cohort (Training set) N = 46	Test Cohort (Testing set) N = 12
Gender [men/women]	8/38	2/10
Age at biopsy [years]	35.50 ± 14.06	38.17 ± 14.06
LN class II/III/IV/V/VI	1/10/21/9/5	0/2/4/3/3
Number of glomeruli in specimen	20.83 ± 10.21	18.08 ± 6.26
Number of totally sclerotic glomeruli	1.61 ± 3.08	2.92 ± 4.68
Number of partially sclerotic glomeruli	3.95 ± 7.79	3.25 ± 3.93
Number of cellular/cellular-fibrotic crescents	1.43 ± 3.07	0.50 ± 1.45
Number of glomeruli with fibrillary necrosis	2.52 ± 4.85	1.58 ± 2.07
Number of glomeruli without changes	1.09 ± 2.50	0.25 ± 0.87
Activity Index	8.99 ± 4.77	8.83 ± 3.51
Chronicity Index	2.95 ± 1.91	3.92 ± 2.78
Interstitial fibrosis [%]	9.02 ± 5.74	13.33 ± 10.73
WBC [10^3/ul]	8.09 ± 3.26	7.34 ± 1.81
NEU [10^3/ul]	5.49 ± 2.61	5.60 ± 2.09
LYM [10^3/ul]	1.90 ± 0.95	1.28 ± 0.71
NLR	3.90 ± 4.06	6.42 ± 5.05
HGB [g/dl]	12.06 ± 1.82	11.26 ± 1.19
PLT [10^3/ul]	238.35 ± 79.15	197.92 ± 70.78
PLR	176.80 ± 147.66	219.48 ± 132.10
ERS [mm/1 h]	37.75 ± 24.92	28.27 ± 15.11
CRP [mg/dl]	4.59 ± 8.67	2.16 ± 2.70
sCr [mg/dl]	1.27 ± 0.69	1.32 ± 0.48
eGFR [ml/min/1.73 m^2^]	67.98 ± 26.65	57.08 ± 26.05
TP [g/dl]	5.35 ± 0.95	5.23 ± 0.89
Albumin [g/dl]	2.87 ± 0.58	2.82 ± 0.52
Total Cholesterol [mg/dl]	274.77 ± 80.20	243.48 ± 89.49
Triglycerides [mg/dl]	202.75 ± 114.55	171.70 ± 68.39
C3 [g/l]	0.86 ± 0.29	0.76 ± 0.29
C4 [g/l]	0.18 ± 0.09	0.16 ± 0.12
ANA (0/1/undefined)	1/39/6	2/9/1
Erythrocyturia [RBC/HPF]	10/19/17	3/4/5
UPCR [mg/mg]	2.91 ± 2.12	4.76 ± 4.83
Target - CR [1/0]	14/32	4/8

**Fig. 1:**
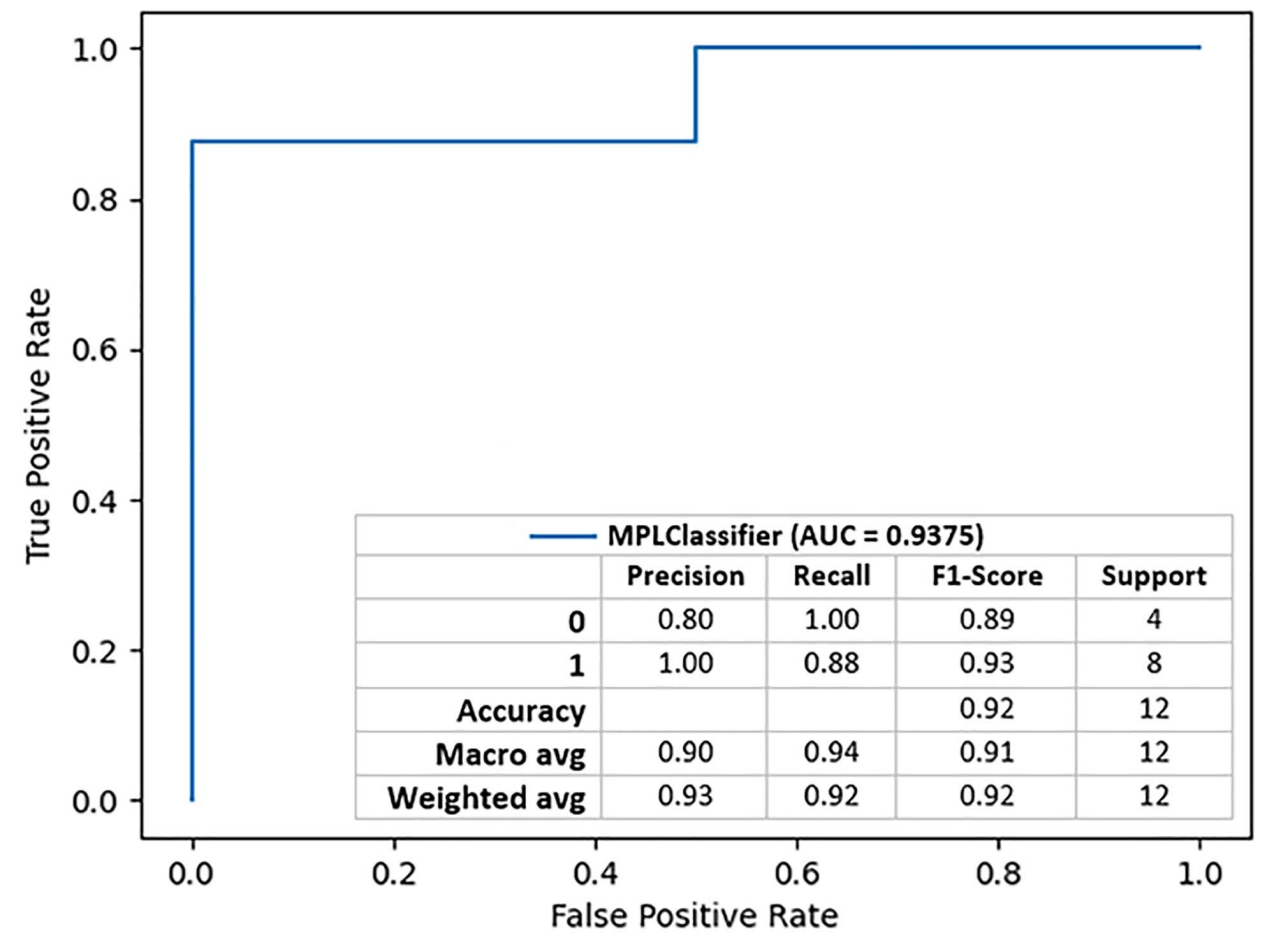
The model with the best performance effectively discriminating the onset and the absence of complete remission in patients with LN. The sensitivity of the model, with respect to the lack of complete remission, makes it a perfect tool for screening people particularly at risk of further complications

A multi-layer perceptron with 40 neurons in the first hidden layer and 45 neurons in the second hidden layer, appeared to be the model with the best performance with AUROC of 0.9375 (0.94), Accuracy of 91.67%, Positive Predictive Value (precision) of 0.9333 and Sensitivity (recall) of 0.9167 (Fig. 1). A similar result was achieved by 2 models built with 8 in the first and 22 in the second layer, and 30 in the first and 41 in the second hidden layer, respectively, but this model turned out to have a lower AUROC of 0.9067.

The best model of artificial neural network achieved 100% precision, for predicting the occurrence of complete remission, in LN from the input variables. Sensitivity 0.88 for a class with complete remission. For the group without complete remission, it achieved 100% sensitivity and 80% positive predictive ability.

The search for the best solution required construction of several models. We made the original assumption about the maximum size of the neural network up to 45 neurons in each of the two layers. In case of failure or unsatisfactory results, we would consider increasing this limit. The obtained result is within the initially assumed limits, i.e., has a relatively low complexity and a superior performance, so it has been considered as an optimal solution combining costs with efficiency.

**Fig. 2:**
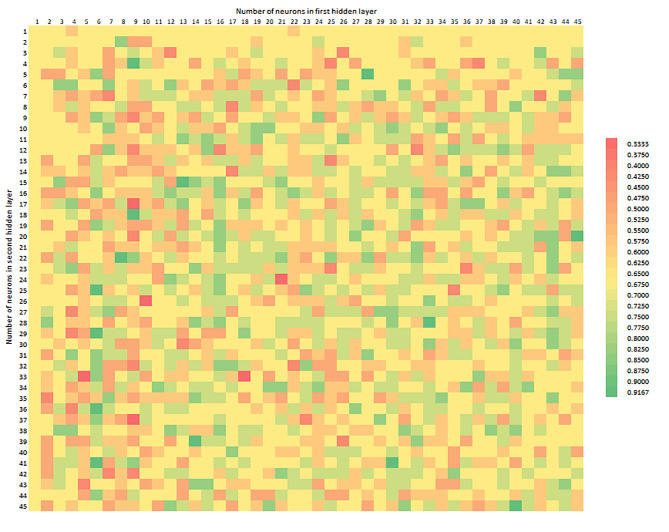
Accuracy depending on the number of neurons in the first (horizontal axis) and second (vertical axis) hidden layer

Figure 2 shows the Accuracy distribution, depending on the number of neurons in the first and second hidden layers. The number of neurons in the first hidden layer is marked on the horizontal axis, whereas the number of neurons in the second hidden layer on the vertical axis. The colour corresponds to an Accuracy value, in the range from 0.3333 to 0.9167, from the worst to the best model constructed. The observation allows to indicate the area where the models were useless and, in the future, it may be possible to construct a metaheuristic, avoiding ineffective solutions and shorten the time of model exploration. The optimal result is a model combining all the parameters as high as possible, considering the costs of its construction and practical application.

**Fig. 3:**
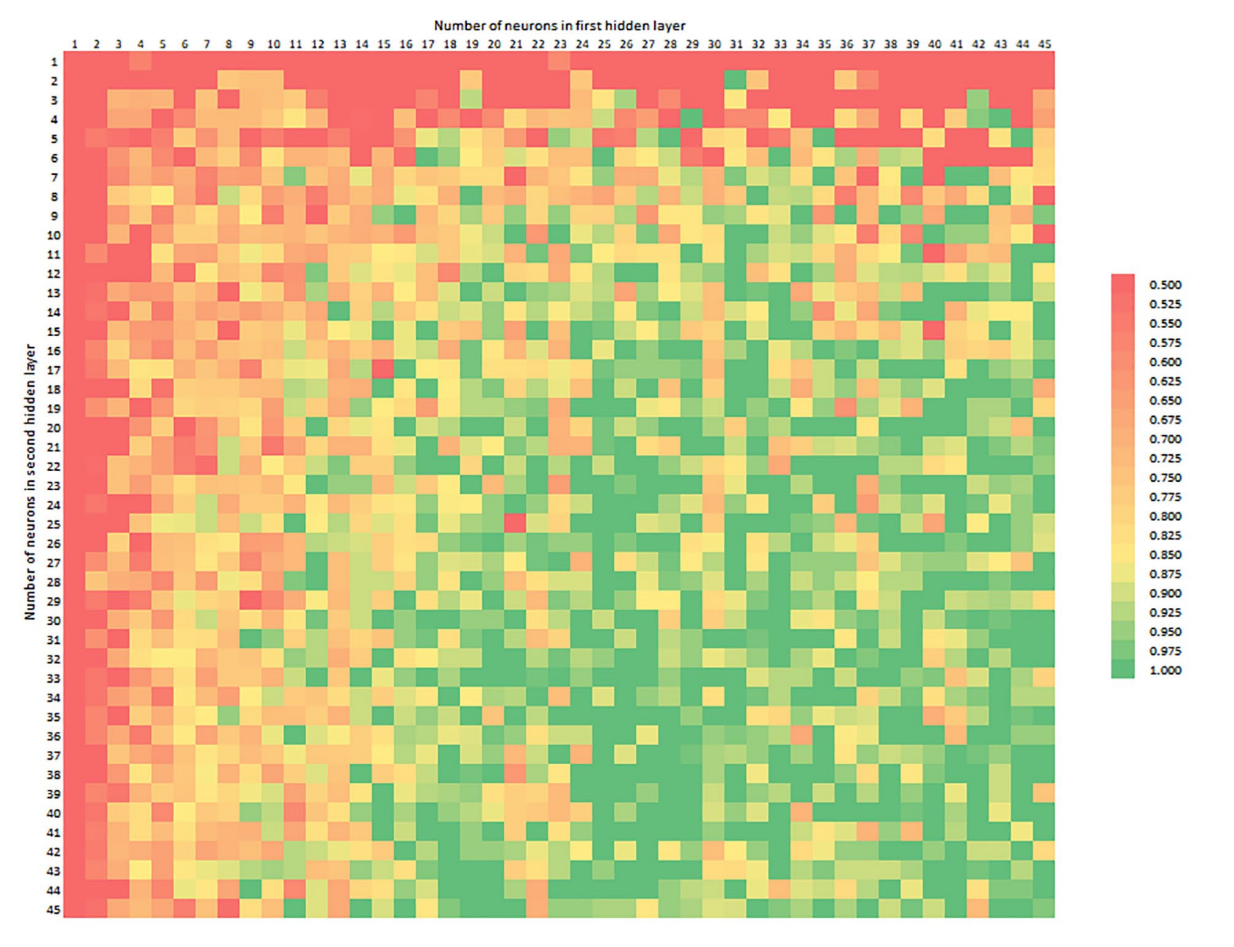
AUROC depending on the number of neurons in the first (horizontal axis) and second (vertical axis) hidden layer

Figure 3 shows the distribution of AUROC, depending on the number of neurons in the first and second hidden layers, with the axes labelled like at Fig. 2. The colour scale starts from 0.500, which is a typical value for a random classifier. The graphic shows an edge area where one layer of the neural network has several neurons and is unable to achieve satisfactory performance regardless of calibrating other hyperparameters or modifying the input variables. Some of the models had AUROC 1.0000, while they had accuracy lower than 0.9. The optimal solution should have both great accuracy and very discriminant power.

**Fig. 4:**
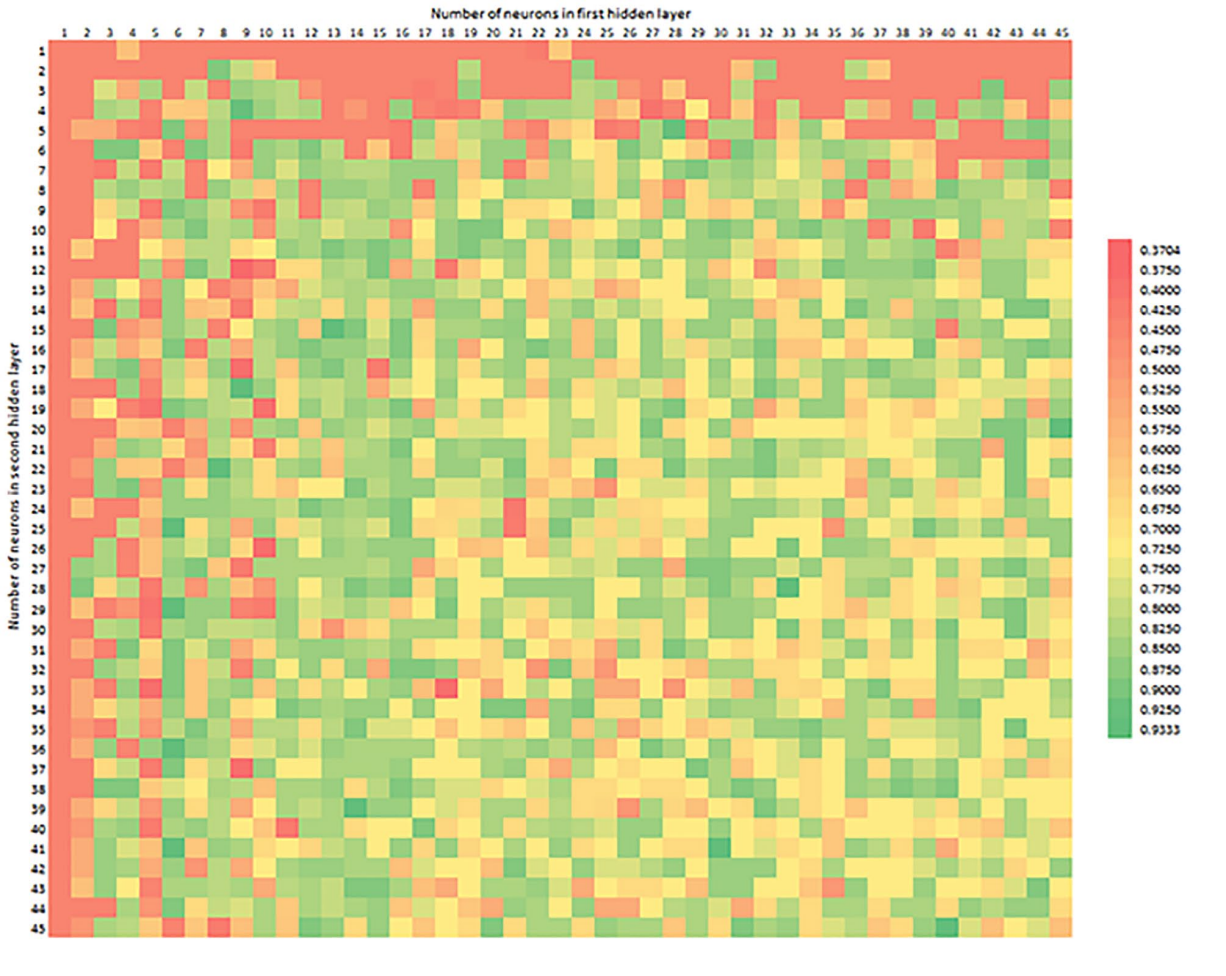
Precision depending on the number of neurons in the first (horizontal axis) and second (vertical axis) hidden layer

Figure 4 shows the precision distribution, depending on the number of neurons, in the corresponding hidden layers. Big data analysis, in combination with a recursive algorithm, allowed to generate various models and select those with higher sensitivity, in relation to the selected weighted average sensitivity target.

**Fig. 5:**
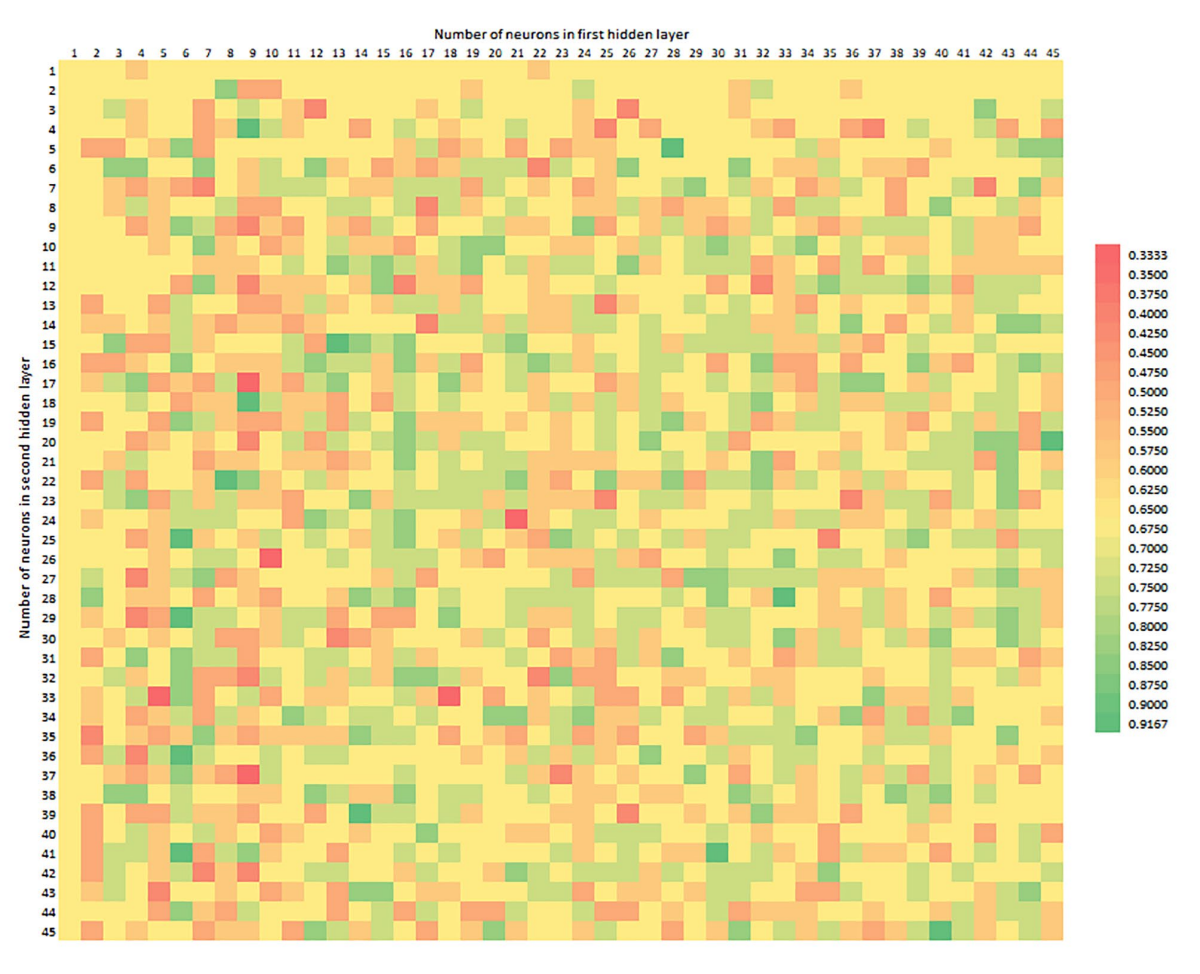
Recall depending on the number of neurons in the first (horizontal axis) and second (vertical axis) hidden layer

Figure 5 shows the Recall distribution, depending on the number of neurons in the corresponding hidden layers. The simplest models, located at the edge of the chart, do not have the worst recall. Due to the slight unbalance of the data set, the average results are recalled around 0.65. The worst outcomes overall and the best ones are scattered inside the graph, showing the complex structure of neural network models.

**Fig. 6:**
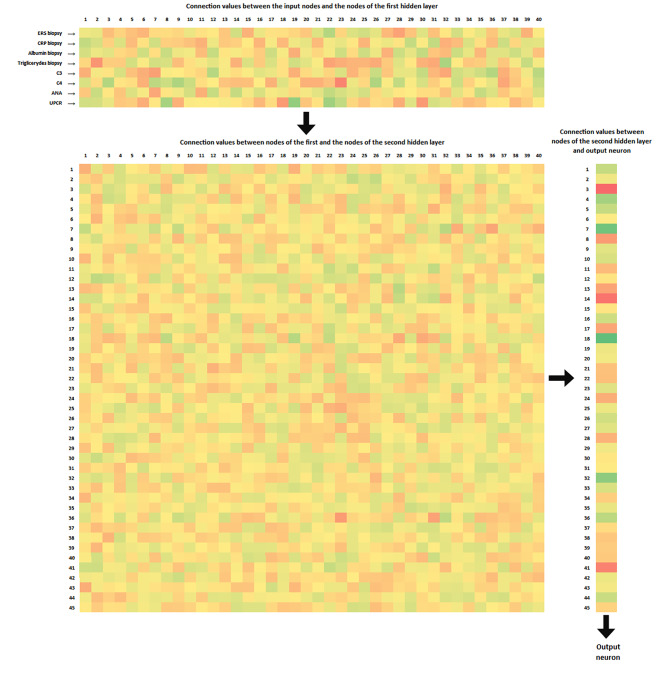
Visualization of connection weights between relevant nodes. For clarity, the weights of links with bias vectors have been omitted. The input variables are the same as the input neurons. Each of the eight input neurons is connected to each of the 40 neurons of the first hidden layer. Each of them is connected to each of the 45 neurons of the second hidden layer. Each of these neurons is connected to an exit neuron. The middle matrix is transposed for graphics

Graphing a neural network, with significant numerical values, may be difficult due to the complexity of the model. Figure 6. shows the matrices, with the values of individual connections between the relevant neurons in specific layers. Our network has the following layers: an input layer with 8 neurons corresponding to specific variables. The first hidden layer consists of 40 neurons. Each of them is connected to the input layer neurons, and the weights of these connections are shown in the upper 8 × 40 matrix in Fig. 6. The second hidden layer consists of 45 neurons, each connected to each of the 40 first hidden layer neurons. The weights of these connections are illustrated by the largest matrix of size 40 × 45 in Fig. 6. The output neuron is connected to each of the 45 neurons of the second hidden layer, and the weights of these connections are shown in the matrix in the right part of the graphic with the size 1 × 45. Due to the transparency of the graphics, we omitted the representation of the so-called vector bias, which are an important element of the network, improving its performance, but we focused on conveying the basic principle of MLP neural network operation.

## Discussion

The input parameters of all neural networks included ERS, CRP, concentrations of serum albumin and triglycerides, complement C3 and C4 levels, presence of ANA, UPCR and data derived from the histopathological examination. Their significance in the assessment of LN progression stay in accordance with the results of studies carried out with implementation of classical statistical analysis. The variables, selected by the computer program, correspond with the conclusions of the research regarding the relationship of individual variables with the severity of the disease.

Simple designs may also achieve a great performance. Liu et al. [11] presented the model, based on UPCR, reaching AUC 0.778, and established with implementation of serum albumin with AUC 0.773. The differences in UPCR and serum albumin were assessed after 3 months follow-up. The cut-off points for change of UPCR and serum albumin concentration were for UPCR ≥ 59%, and for serum albumin ≥ 32.9 g/l, respectively and allowed to predict remission of LN, at sixth month follow-up. The level of C3 complement component, at the time of follow-up, allowed the prediction of LN remission, with an AUC of 0.701. Similar parameters were demonstrated in our study as reliable markers in prediction of LN remission. Chen et al. [12] obtained a design with AUC 0.819, in the validation cohort, using 59 input variables. Most of them were assessed at the point of remission. The simplified Cox risk score model implemented 6 variables, derived from initial features set, and subsequently employed to assess the risk of renal flare with AUC of 0.746. Tang et al. [13] investigated clinical indices with respect to machine learning techniques and achieved an accuracy of 40.1–56.2% in depending on the predicted LN class.

Adamichou et al. developed a more complex model, capable of recognizing LN with accuracy of 97.9% [14]. In our work, we tried to avoid too obvious variables, directly leading to a given result, so we avoided differentiating the healthy versus sick ones as a trivial issue. A comparative solution, with a list of several machine learning techniques, was presented by Helget et al. [15], with results of AUC 0.800 for Random Forest Classifier, using 4 variables: chronicity score, intestinal inflammation, UPCR and WBC. An Artificial neural network design based on activity score, chronicity score, intestinal fibrosis, intestinal inflammation and UPCR, achieved AUC of 0.775.

Regarding renal histopathology, as a crucial factor for the clinical management and outcome of patients with LN, it is worth to mention that deep learning-based AI procedure was also tested for automatic assessment of glomerular pathological findings in LN [16]. The main motive for the development of such an arrangement was an unsatisfactory inter-pathologist agreement. Deep convolutional neural network-based system detected and classified glomerular pathological findings in LN (dataset of 349 renal biopsy whole-slide images). Authors suggested that deep learning is a feasible assistive tool for the objective and automatic assessment of pathological LN lesions: at the per-patient kidney level, the model achieved a high agreement with nephropathologist (linear weighted kappa: 0.855, 95% CI; quadratic weighted kappa: 0.906, 95% CI).

One of the most serious limitations of our study was the small size of the examined population. This was a was single-centre study, conducted on an ethnically homogeneous population. The obtained models are scalable, and, in the future, we hope to test them on a larger group. A particular advantage is the use of neural networks that may be retrained on a smaller group of samples, called partial fitting. Machine learning is not a technique, which may be comparable between centres, as are the classic analysis, based on odds ratio and survival models. Despite the insight into the mechanism of operation, we were not able to draw greater conclusions without an in-depth mathematical and computer analysis of the algorithm, requiring knowledge and experience in computer science. The MLP neural network, on the other hand, is a practical tool that may be used in clinical practice after appropriate calibration for the population.

## Conclusion

The use of an artificial neural network, learned even on a small patient cohort, allows the construction of a predictive model, with good or very good performance. A huge advantage is the ability to scale models to larger and more diverse populations and over-write the values stored in the network structure with partial fitting. We emphasize the possibility of using this solution in a pilot program after conducting further observations on a larger research group.

## Data Availability

The datasets used and/or analysed during the current study are available from the corresponding author on request.

## List of abbreviations

ALB: serum albumin
ANA: antinuclear antibodies,
C3: complement component 3
C4: complement component 4
CRP: C-reactive protein
eGFR: estimated glomerular filtration rate
ERS: erythrocytes sedimentation rate
HGB: haemoglobin
LN: lupus nephritis
LYM: lymphocytes
MLP: multi-layer perceptron
NEU: neutrophils
NLR: neutrophil-to-lymphocyte ratio
PLR: platelets to lymphocyte ratio
PLT: platelets
sCr: serum creatinine concentration.
SLE: systemic lupus erythematosus.
TP: total protein concentration.
UPCR: urine protein to creatinine ratio.
WBC: white blood cells.
